# Evaluation of a novel lyophilized-pellet-based 2019-nCoV nucleic acid detection kit for the diagnosis of COVID-19

**DOI:** 10.1371/journal.pone.0292902

**Published:** 2023-10-25

**Authors:** Yiyuan Xu, Tian Xu, Shaoting Chen, Huakang Yao, Yuxiang Chen, Yanfen Zeng, Falin Chen, Guanbin Zhang

**Affiliations:** 1 Research and Development Department, Fujian CapitalBio Medical laboratory, Fuzhou, Fujian, China; 2 Clinical Laboratory, Fujian Provincial Hospital, Fuzhou, Fujian, China; 3 Medical Department, Fujian Provincial Yongtai County Hospital, Fuzhou, Fujian, China; 4 Fujian Provincial Center for Clinical Laboratory, Fujian Provincial Hospital, Fuzhou, Fujian, China; 5 Research and Development Department, National Engineering Research Center for Beijing Biochip Technology, Beijing, China; 6 Department of Laboratory Medicine, Fujian Medical University, Fuzhou, Fujian, China; Huadong Research Institute for Medicine and Biotechniques, CHINA

## Abstract

The coronavirus disease 2019 (COVID-19) caused by severe acute respiratory syndrome coronavirus 2 (SARS-CoV-2) has swept the world and poses a serious threat to human health. In the post-pandemic-era, we must remain vigilant against the co-infection of SARS-CoV-2 and other respiratory viruses. More accurate and convenient detection methods are required for the diagnosis of SARS-CoV-2 due to its prolonged existence. In this study, the application value of a novel lyophilized-pellet-based 2019-nCoV nucleic acid diagnostic kit (PCoV-Kit) was evaluated by comparing it with a conventional liquid diagnostic kit (LCoV-Kit). We assessed the sensitivity, precision, accuracy, specificity, and amplification efficiency of PCoV-Kit and LCoV-Kit using diluted SARS-CoV-2 RNA reference materials. The results showed that both kits had high sensitivity, precision, accuracy, and specificity. A total of 2,033 oropharyngeal swab specimens collected during mass screening in Fuzhou in December 2022 were applied for the consistency analysis of the two reagents. In the detection of clinical oropharyngeal swab specimens, although the positive rate of PCoV-Kit (19.28%) was slightly lower than that of LCoV-Kit (20.86%), statistical analysis demonstrated a high degree of consistency between the test results obtained using both kit (χ^2^ = 1.57, *P*>0.05; Kappa coefficient = 0.90, 95%CI: 0.88–0.93). In conclusion, the use of lyophilized PCoV-Kit provides a non-inferior assay for the diagnosis of COVID-19.

## Introduction

In the past three years, coronavirus disease 2019 (COVID-19), caused by severe acute respiratory syndrome coronavirus 2 (SARS-CoV-2), created an unprecedented crisis for global public health and safety [1]. As of 31 May 2023, 767,364,883 confirmed cases of COVID-19, including 6,938,353 deaths, reported to WHO.

Accurate, rapid, reliable, and convenient diagnosis tests are crucial for the control and treatment of SARS-CoV-2 infection. Laboratory assays for diagnosing SARS-CoV-2 infection include nucleic acid amplification testing (NAAT), antigen tests, and serologic testing [2]. NAAT and antigen tests are generally used for early detection for SARS-CoV-2 infection around the time of symptom onset, while serologic testing is used 3 weeks after infection for surveillance purposes [3,4]. As reported in a network meta-analysis of diagnostic test accuracy, NAAT had a sensitivity of 0.93 (95%CI: 0.88–0.96) and specificity of 0.98 (95%CI: 0.97–0.99), while antigen testing would lead to higher false negatives (sensitivity of 0.75, 0.70–0.79; specificity of 0.98, 0.97–0.99) [5]. NAAT method contains reverse-transcriptase ploymerase chain reaction (RT-PCR) and isothermal nucleic acid amplification (NAA) [2]. RT-PCR is considered to be the gold standard [6].

During the COVID-19 pandemic, the transmissibility of SARS-CoV-2 has gradually increased on the rise due to its ongoing mutations [7]. SARS-CoV-2 is predicted to coexist with humans for an extended period of time [8]. Studies indicated that SARS-CoV-2 frequently co-infected with influenza viruses, respiratory syncytial virus, adenoviruses, or dengue virus, leading to more severe clinical symptoms and an increase in mortality [9,10]. Since SARS-CoV-2 infection shares overlapping clinical symptoms with other viral infections mentioned above, accurate diagnosis plays a crucial role in guiding clinical treatments [9].

Currently, the commonly used RT-PCR reagents are liquid reagents requiring fresh preparation, which possesses several drawbacks: (i) liquid reagents require strict cold chain transportation and storage, (ii) repeated freeze-thaw cycles can lead to the degradation of DNA or the inactivation of amplification enzyme, (iii) mistakes in final concentration or pipetting errors during reaction mix preparation may result in inaccurate and delayed results [11,12]. In contrast, ready-to-use lyophilized reagents simplify the transportation and storage requirements and minimize user interference, making them a more cost-effective and convenient option for diagnosis purposes. It has previously been demonstrated that the lyophilized reagents are reliable and accurate detection reagents in the diagnosis of chikungunya virus, Rift Valley fever phlebovirus, foot-and-mouth disease virus, and avian influenza viruses [12–15].

In this study, we compared a a novel lyophilized-pellet-based 2019-nCoV nucleic acid diagnostic kit (PCoV-Kit) with a widely used commercial liquid diagnostic kit (LCoV-Kit), assessing its sensitivity, precision, accuracy, amplification efficiency, and specificity. In addition, we evaluated the consistency of the two reagents in detecting clinical specimens.

## Material and methods

### Reagents

The following reagents were used in this study: SARS-CoV-2 RNA reference material (Guangzhou BDS Biological Technology, China, Cat. #BDS-BW-117); 2019-nCoV-RNA liquid internal quality control (Guangzhou BDS Biological Technology, China, Cat. #BDS-IQC-304); sample preservation solution (CapitalBio Technology, China, Cat. #3010202033); nucleic acid (DNA/RNA) extraction or purification kit (Sansure Biotech, China, Cat. #SE40015); novel coronavirus (2019-nCoV) nucleic acid diagnostic kit (PCR-Fluorescence Probing) (Sansure Biotech, China, Cat. #010101510, hereinafter referred to as LCoV-Kit; CapitalBio Technology (Chengdu), China, Cat. #3010202044, hereinafter referred to as PCoV-Kit).

SARS-CoV-2 RNA reference material and 2019-nCoV-RNA liquid internal quality control contained the complete sequence of ORF 1a, ORF 1b, N, E, S, and M genes of SARS-CoV-2, along with the mutation sites of Omicron and Delta variants. To generate lentivirus particles, target RNA vectors and lentivirus packaging vectors were co-transfected into 293T cells. The lentivirus particles were subsequently diluted to the desired concentration. The N, E, and ORF1ab genes in the SARS-CoV-2 RNA reference material were quantified using droplet digital PCR. SARS-CoV-2 RNA reference material is registered in the National Reference Material Center with the registration number GBW(E)091132.

LCoV-Kit and PCoV-Kit detect two conserved target sequences in the SARS-CoV-2, namely open reading frame (ORF) 1ab and nucleocapsid-encoding protein (N) genes. Both kits have obtained the medical device registration certificate from China Food and Drug Administration (Certificate No. 20203400064 and 20223401469, respectively).

### Clinical specimens

Between December 1 and December 22, 2022, we randomly selected approximate 100 oropharyngeal swab specimens per day from the remaining samples collected during the COVID-19 mass screening in Fuzhou, resulting in a total of 2,033 specimens. The study was approved by the ethics review committees of Fujian provincial hospital, Chinese (K2022-11-005), and all procedures were performed in accordance with the relevant guidelines and regulations. Oral consents were obtained from all participants.

### Nucleic acid extraction and PCR amplification

Nucleic acid was extracted from 300 μL sample using Natch 96B automatic nucleic acid extractor (Sansure Biotech) according to the instrument instruction manual. When using LCoV-Kit for amplification, 20 μL RNA extract was mixed with 26 μL of reaction buffer and 4 μL of enzyme mix to a 50-μL final volume. When using PCoV-Kit, 20 μL RNA extract was directly added into the PCR tube containing lyopholized pellet and gently mixed to form a final volume of 20 μL. For qualitiy control, a positive control, borderline positive control, and two negative controls were induced in each batch. The positive control was obtained from the kit. The borderline positive control was prepared by diluting the 2019-nCoV-RNA liquid internal quality control, which had an initial concentration of 1000 copies/mL, in a two-fold manner. The negative controls were saline and the negative control provided in the kit, respectively. PCR amplification was performed on SLAN-96S fluorescence quantitative PCR instrument (Shanghai Hongshi Medical Technology, China). The viral target included open reading frame 1ab (ORF1ab) and nucleocapsid protein (N). Ct value were calculated for both reference gene and target genes with auto-baseline and auto-threshold. The results are defined positive if Ct value <40, otherwise negative. Samples are identified as positive when the N gene and ORF1ab gene are both positive.

### Sensitivity evaluation

SARS-CoV-2 RNA reference material BDS-BW-117 (2.0×10^5^ copies/mL) was diluted with sample preservation solution to 150 copies/mL, the limit of detection (LOD) stated in the manual of PCoV-Kit. The nominal detection limit of LCoV-Kit was 200 copies/mL. The extracted RNA was tested 20 times in duplicate using PCoV-Kit and LCoV-Kit, respectively, to verify the sensitivity. The kit was considered to pass the verification if the coincidence rate of positive results was ≥95%.

### Precision evaluation

SARS-CoV-2 RNA reference material BDS-BW-117 (2.0×10^5^ copies/mL) was diluted with sample preservation solution to the concentration of 300 copies/mL (C300) and 500 copies/mL (C500), respectively. RNA extracted from C300 and C500 samples was tested 10 times in duplicate with PCoV-Kit and LCoV-Kit, respectively. The mean and standard deviation (SD) of Ct values of 10 test data were calculated, and coefficient of variation (CV) value was obtained according to the following formula: CV = SD/M*100%. CV ≤5% was considered to be stable.

### Accuracy evaluation

We selected 10 positive samples (5 each for C300 and C500) and 10 negative samples (sample preservation solution) as the sample pool for the accuracy evaluation. All analyses were performed after random consecutive numbering of samples, which was only revealed after finishing all analyses. The test results were compared with the expected results, and the negative, positive and total coincidence rates of PCoV-Kit and LCoV-Kit were calculated. Evaluation of the specificity of the assays was described in S1 Appendix.

### Amplification efficiency measurement

The SARS-CoV-2 RNA reference material (2.0×10^5^ copies/mL) was diluted 1:10 in sample preservation solution to form concentration gradients: SE5 (2.0×10^5^ copies/mL), SE4 (2×10^4^ copies/mL), SE3 (2×10^3^ copies/mL), and SE2 (2×10^2^ copies/mL). RNA extracted from SE5-SE2 samples was tested 5 times in duplicate with PCoV-Kit and LCoV-Kit, respectively. An amplification standard curve was plotted of Log (concentration) versus Ct value and the slope (K) was derived. The amplification efficiency (E) was calculated according to the formula: E = 10^−1/K^-1.

### Testing of clinical specimens and statistical analysis

RNA of 2,033 oropharyngeal swab specimens was extracted as described above and detected with PCoV-Kit and LCoV-Kit, respectively. Statistical analysis was performed using SPSS and statistical significance was assumed at *p* < 0.05. Normal distribution was reported as means with SD. Pearson χ2 was used to compare the positive test results. Sensitivity, specificity, predictive value of positive test (PVP), predictive value of negative test (PVN) and Cohen’s kappa coefficient (κ) were calculated using DAG_Stat (https://biostats.com.au/DAG_Stat/). κ was used to assess the consistency of the test results. When κ value is 0–0.40 indicated poor agreement, 0.41–0.60, moderate agreement, 0.61–0.80, good agreement, and >0.80, very good agreement. Box plots, generated with Origin, was used to show the distribution of Ct values and test results using different kits.

## Results

### Sensitivity evaluation

When 150 copies/mL of SARS-CoV-2 reference material was used for testing, the positive detection rates of PCoV-Kit was 100%, and the detection rates of single target genes (ORF1ab and N gene) were both 20/20, which met the nominal LOD of PCoV-Kit manufacturer. The LCoV-Kit, with a nominal LOD of 200 copies/mL, demonstrated a detection rate of 100% comparable to that of PCoV-Kit. The sensitivity test results are shown in Table 1, while the corresponding raw data can be found in S1 Table.

**Table 1:** Sensitivity evaluation of PCoV-Kit and LCoV-Kit (*x*±*s*, *n =* 20).

	N gene	ORF1ab gene
**PCoV-Kit**	33.48±0.49	37.31±0.49
**LCoV-Kit**	35.24±0.43	38.60±0.61

### Precision evaluation

According to the analysis, the CV of the test results of C300 (300 copies/mL) and C500 (500 copies/mL) samples using PCoV-Kit and LCoV-Kit was less than 3%, indicating good precision. Especially, when the two reagents were used to detect C500, the CV of both two target genes was less than 1%, indicating that the two reagents had better precision and stability when detecting high concentration samples. The results of precison evaluation are presented in Table 2, while the corresponding raw data can be found in S2 Table.

**Table 2:** Precision evaluation of PCoV-Kit and LCoV-Kit (*x*±*s*, *n =* 10).

Reagents	C300		C500	
	Mean Ct value	CV	Mean Ct value	CV
**N gene**				
**PCoV-Kit**	31.96±0.19	0.60%	29.59±0.11	0.37%
**LCoV-Kit**	33.76±0.37	1.09%	31.04±0.27	0.86%
**ORF1ab gene**				
**PCoV-Kit**	36.13±0.86	2.39%	32.48±0.12	0.35%
**LCoV-Kit**	37.18±0.40	1.08%	33.23±0.15	0.44%

### Accuracy evaluation

Blind test of SARS-CoV-2 in 10 randomly numbered samples showed that the total coincidence rate of PCoV-Kit and LCoV-Kit was 100%, indicating that these two reagents had good accuracy. The corresponding raw data are presented in S3 Table.

### Amplification efficiency measurement

**Fig 1:**
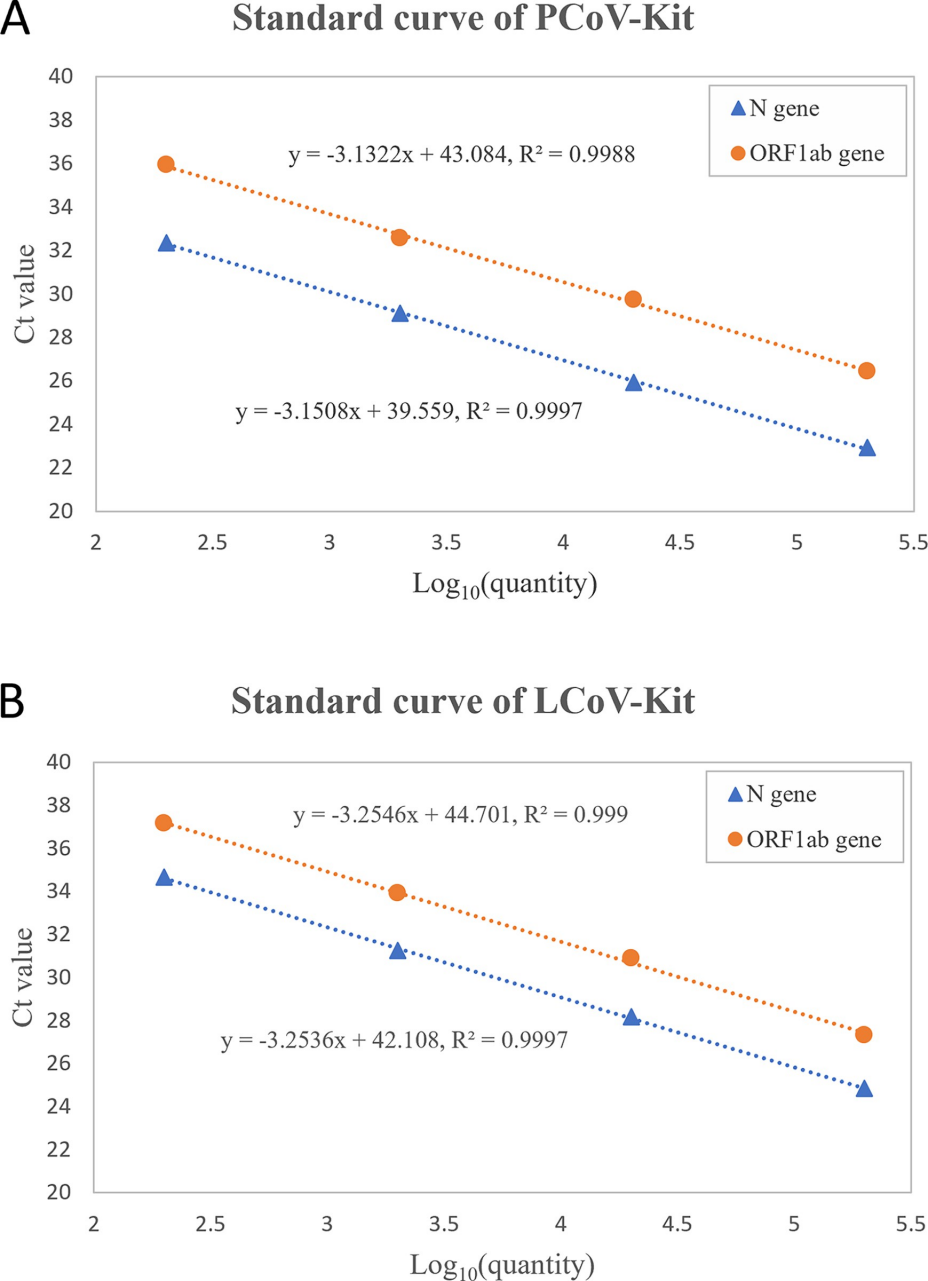
Comparision of standard curves for two reagents. (A) Standard curve of PCoV-Kit. The slope of the N gene curve was -3.1508, and the *R*^*2*^ was 0.9997. The slope of the ORF1ab gene curve was -3.1322, and the *R*^*2*^ was 0.9988. (B) Standard curve of LCoV-Kit. The slope of the N gene curve was -3.2536, and the *R*^*2*^ was 0.9997. The slope of the ORF1ab gene curve was -3.2546, and the *R*^*2*^ was 0.999. Each dot corresponds to the mean value of five replicates.

Ten-folds serial dilutions of SARS-CoV-2 RNA reference material ranging from SE5 (2.0×10^5^ copies/mL), SE4 (2×10^4^ copies/mL), SE3 (2×10^3^ copies/mL), to SE2 (2×10^2^ copies/mL) were used to generate the standard curve. The standard curve was drawn according to the quantity and Ct value of the samples. The results are shown in Table 3 and Fig 1, and the corresponding raw data are presented in S4 Table. The amplification efficiency of N gene and ORF1ab gene for PCoV-Kit were 107.7% and 108.9%, respectively, while they were both 102.9% for LCoV-Kit, indicating that the amplification efficiency of LCoV-Kit was closer to the optimal state. The correlation coefficient (*R*^*2*^) for each standard curve was above 0.99, suggesting good linearity.

**Table 3:** Statistics of amplification efficiency measurement.

Reagents	SE5	SE4	SE3	SE2	Amplification efficiency
**N gene**					
**PCoV-Kit**	22.92±0.04	25.93±0.03	29.12±0.07	32.36±0.15	107.7%
**LCoV-Kit**	24.85±0.26	28.19±0.21	31.26±0.16	34.67±0.69	102.9%
**ORF1ab gene**					
**PCoV-Kit**	26.46±0.07	29.74±0.13	32.56±0.10	35.96±0.36	108.6%
**LCoV-Kit**	27.33±0.09	30.90±0.12	33.93±0.18	37.16±0.43	102.9%

### Testing of clinical specimens and statistical analysis

To evaluate the performance of two reagents, a total of 2,033 oropharyngeal swab specimens were collected during mass screening in Fuzhou in December 2022, and detected by PCoV-Kit and LCoV-Kit, respectively. The raw data of specimens are presented in S5 Table. As shown in Table 4, the average age of the subjects was 39.3±13.5 years (range, 1–83 years), with 63.1% of the population being male and 36.9% female.

**Table 4:** Demographic characteriscs of clinical specimens.

**Characteristics**	N = 2,033
**Male–n / total N (%)**	1282 (63.1)
**Age—median years (min-max)**	39.3 (1–83)
**age groups–n (%)**	
**[1–11]**	50 (2.5)
**(11–21]**	148 (7.3)
**(21–31]**	354 (17.4)
**(31–41]**	593 (29.2)
**(41–51]**	495 (24.3)
**(51–61]**	303 (14.9)
**(61–71]**	71 (3.5)
**>71**	19 (0.9)

Overall, 392 (19.28%) were tested positive by PCoV-Kit and 424 (20.86%) by LCoV-Kit (Table 5). PCoV-Kit correctly classified 96.85% (95%CI: 96.00%-97.57%) of all samples with a sensitivity of 88.68% (95%CI: 85.27%-91.53%), specificity of 99.01% (95%CI: 98.39%-99.43%), PVP of 95.92% (95%CI: 93.46%-97.65%), and PVN of 97.07% (95%CI: 96.14%-97.84%).

**Table 5:** The consistency of test results by PCoV-Kit and LCoV-Kit.

Samples		PCoV-Kit		Total
		Positive	Negative	
**LCoV-Kit**	**Positive**	376	48	424
	**Negative**	16	1,593	1,609
**Total**		392	1,641	2,033

A chi-square test performed by SPSS 22.0 confirmed there was no significant difference in the positive detection rate of PCoV-Kit and LCoV-Kit (χ^2^ = 1.57, *P*>0.05). The Kappa coefficient was 0.90 (95%CI: 0.88–0.93), indicating a good consistency between the two reagents.

**Fig 2:**
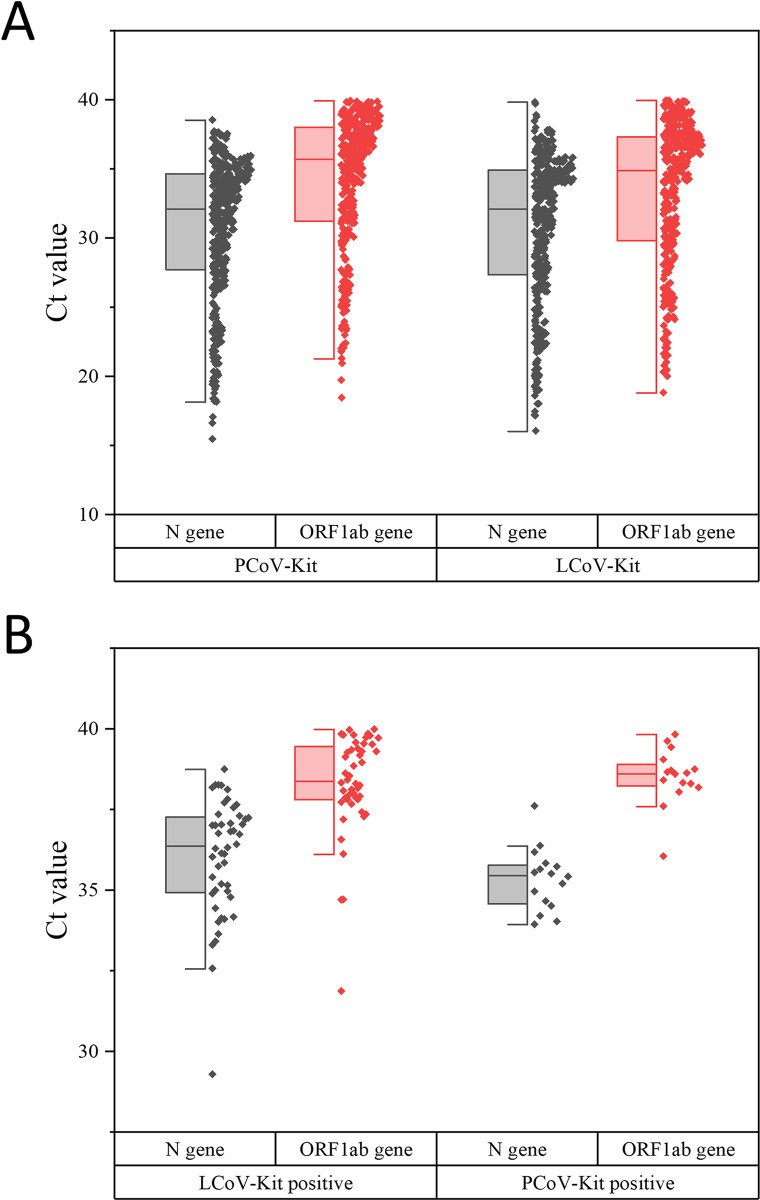
Distribution of Ct values comparison. (A) Ct value distribution of oropharyngeal swab specimens that tested positive using both PCoV-Kit and LCoV-Kit (*n* = 376). (B) Ct value distribution of oropharyngeal swab specimens exhibiting inconsistent results, such as those that were positive for LCoV-Kit but negative for PCoV-Kit (*n* = 48) or vice versa (*n* = 16).

Furthermore, to assess the Ct value distribution and quartiles, boxplots were shown in Fig 2. We calculated the Ct value of 376 oropharyngeal swab specimens that tested positive with both LCoV-Kit and PCoV-Kit, and the statistics are presented in S6 Table. The Ct values for N gene were 30.81±5.30 and 30.79±5.00 with LCoV-Kit and PCoV-Kit, respectively. For ORF1ab gene, the Ct value were 33.27±5.19 and 34.11±4.89 with LCoV-Kit and PCoV-Kit, respectively. The performance of both reagents was comparable. Among the 64 samples with inconsistent test results, 48 were identified as positive by LCoV-Kit but negative (*n* = 18) or single-gene positive (*n* = 30) by PCoV-Kit. The statistical data of the 64 samples are presented in S7 Table. These samples had a mean Ct value of 36.03±1.83 for the N gene and 38.30±1.56 for the ORF1ab gene. In addition, 18 samples were identified as positive by PCoV-Kit but negative (*n* = 4) or single-gene positive (*n* = 12) by LCoV-Kit. These samples had a mean Ct value of 35.34±0.96 for the N gene and 38.51±0.87 for the ORF1ab gene.

## Discussion

As a highly infectious virus, SARS-CoV-2 triggered a global pandemic and posed a threat to public health. A study showed that population density is one of the main determinants of SARS-CoV-2 infection [16]. Meanwhile, the elderly population were more susceptible to SARS-CoV-2 infection and experienced a less effective immune response after vaccination [17–19]. As the most populous country in the world, China is densely populated and facing the largest and fastest growth in population aging [20]. Although widespread vaccination has reduced the risk of SARS-CoV-2 infection and the morality of patients, attention still needs to be paid to co-infection with other viruses, such as influenza viruses, which often leads to more severe clinical symptoms and increased morality [9,10,19]. Additionally, it is imperative for the government and medical institutions to conduct ongoing surveillance of vulnerable and high-risk populations, such as the elderly. Under the circumstances, the application of precise, rapid, and convenient detection reagents is crucial for clinical diagnostics.

When employing conventional liquid RT-PCR reagents, trained personnel and a separated laboratory for reagent preparation are requisite [21]. In addition, user intervention, such as pipetting errors and final concentration mistakes, may impede the accuracy of test results [11,12]. The "Guidelines for the Management of Cold Chain Transportation and Storage of Medical Devices" issued by the China Food and Drug Administration require continuous temperature monitoring during the transportation and storage of in vitro diagnostic reagents to ensure compliance with requirements. Therefore, the lower the storage temperature of the reagents, the higher the transportation and storage costs incurred. The aforementioned limitations can be overcome by using ready-to-use lyophilized reagents, which simplify reagents storage requirements, reduce transportation cost, and avoiding repeated freeze-thaw of reagents. This makes lyophilized reagents a viable substitute for conventional liquid reagents.

**Fig 3:**
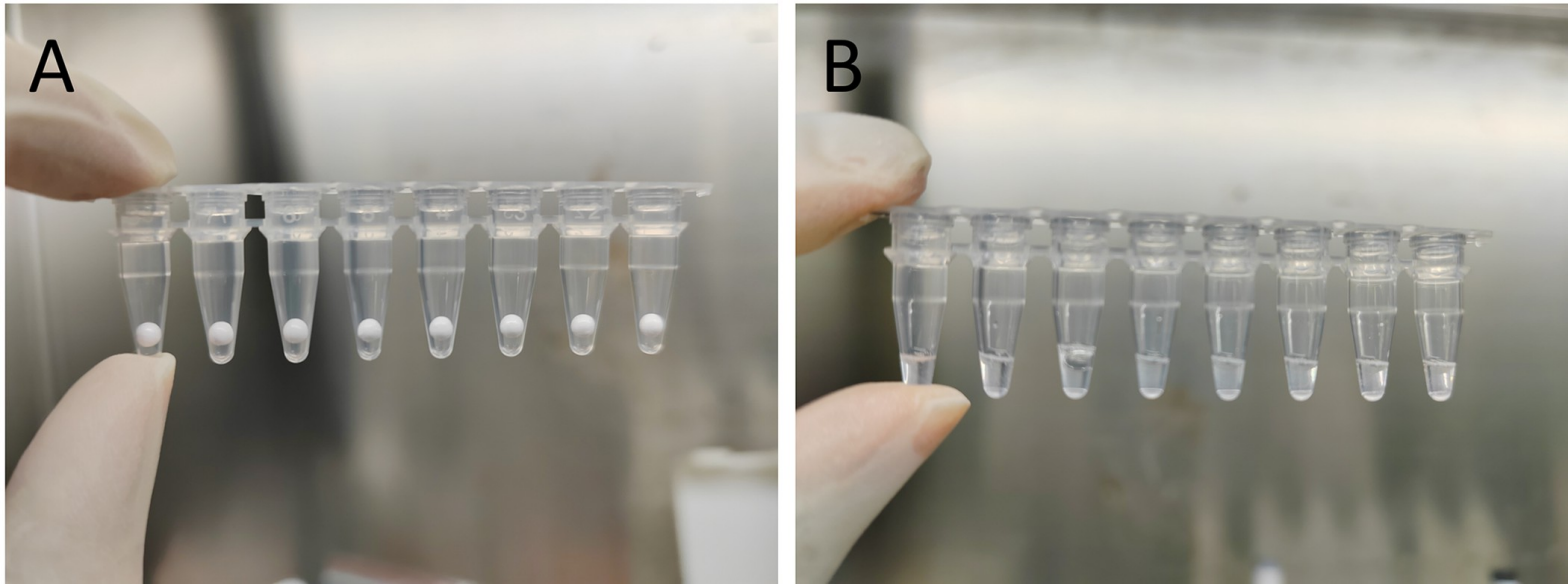
Photograph of lyophilized-pellet-based 2019-nCoV nucleic acid diagnostic kit (P-CoV-Kit). (A) The appearance of the lyophilized pellets. (B) The pellets were dissolved upon addition of nucleic acid samples.

In this study, we compared the performance of a novel lyophilized-pellet-based 2019-nCoV nucleic acid diagnostic kit (PCoV-Kit) with a widely used commercial liquid diagnostic kit (LCoV-Kit) with the aim of providing clinical reference for selecting appropriate detection reagents. The amplification reagents of PCoV-Kit were lyophilized into pellets and encapsulated in individual PCR tubes as pellets. Upon usage, the pellets could be easily dissolved by adding nucleic acid samples to the PCR tubes and shaking. Fig 3 illustrates both the appearance of the pellets and their dissolved form.

In the sensitivity evaluation, both kits exhibited excellent sensitivity. PCoV-Kit showed positive results in all 20 tests with a sample concentration of 150 copies/mL, meeting the nominal LOD in manufacture. Although LCoV-Kit has a nominal LOD of 200 copies/mL, it still achieved a 100% (20/20) positive detection rate for samples, albeit with Ct value closed to the negative cut-off value (38.60±0.61). This result indicatedthat the sensitivity of PCoV-Kit and LCoV-Kit were comparable. Both kits also demonstrated good precision and accuracy, and there was no cross-reactivity observed with prevalent respiratory pathogens. In terms of amplification efficiency evaluation, PCoV-Kit and LCoV-Kit both exhibited excellent linearity. The amplification efficiency for the N gene and ORF1ab gene using PCoV-Kit were 107.7% and 108.9%, respectively, while they were both 102.9% when using LCoV-Kit. With an amplification efficiency ranging from 90% to 110%, the PCR assay is generally recognized as a hallmark of quality capable of producing dependable data [22]. When the amplification efficiency approaches 100%, it indicates that the amplification process is approaching an ideal state [23]. Thus, both reagents were adequate for qualitative diagnosis in clinical settings, although LCoV-Kit performed a better amplification performance than PCoV-Kit.

When 2,033 oropharyngeal swab specimens were tested using both kits simultaneously, the positive rate of PCoV-Kit was 19.28%, which was slightly lower than that of LCoV-Kit (20.86%). This finding contradicted the superior sensitivity evaluation performance of PCoV-Kit over LCoV-Kit, possibly due to the presence of PCR amplification interference substances in clinical specimens, such as food residues or oral epithelial cells that subsequently lyse and release large amounts of cellular components [24]. Therefore, the anti-interference capacity of PCoV-Kit was weaker than that of LCoV-Kit, possibly attributed to the fact that the liquid component in the amplification system of PCoV-Kit solely originates from samples (20 μl), whereas LCoV-Kit’s amplification system contains a buffer system of 30 μl in addition to 20 μl samples. The positive rates of the two kits were statistically analyzed, and no significant difference was found (χ^2^ = 1.57, *P*>0.05). The Kappa coefficient was 0.90 (95%CI: 0.88–0.93), indicating a high level of consistency in the test results of PCoV-Kit and LCoV-Kit. Additionally, the distribution of Ct values of samples that tested positive with both kits also exhibited a high degree of consistency, as illustrated in Fig 2A.

We examined the Ct value distribution of samples with inconsistent test results (positive versus negative/single positive) and found that they were mainly concentrated in the range of 35~40, which falls within the borderline positive region of detection [25]. At this point, the copy number of target genes was small, close to or below the LOD of reagents, making false negatives more likely. As indicated by the list of discrepancies in test results (S8 Table), a majority of the cases were single positive (PCoV-Kit, 30/48; LCoV-Kit, 12/16). Despite being deemed negative, these samples could prompt testing personnel to employ more sensitive detection reagents for retesting purpose, thereby ensuring the accuracy of test results.

In conclusion, PCoV-Kit, as a novel lyophilized-pellet-based SARS-CoV-2 detection kit, obviates the need for cold chain transportation and freezing storage (Its storage temperature is 2~8°C), eliminates the requirement for pre-reagent preparation, thereby saving labor, transport and storage cost and laboratory space, and reduce the time required for detection. In our testing, the detection performance of PCoV-Kit, including sensitivity, precision, and accuracy, can match or exceed that of conventional liquid reagent (LCoV-Kit). In the detection of clinical oropharyngeal swab specimens, although its anti-interference capacity was inferior to that of LCoV-Kit, the reliability of its test results remained satisfactory. In the post-pandemic era, lyophilized PCR reagents are more suitable for COVID-19 diagnosis use.

## Supporting information

S1 Table Raw data for the sensitivity evaluation of PCoV-Kit and LCoV-Kit. (XLSX)

S2 Table Raw data for the precision evaluation of PCoV-Kit and LCoV-Kit. (XLSX)

S3 Table Raw data for the accuracy evaluation of PCoV-Kit and LCoV-Kit. (XLSX)

S4 Table Raw data for the amplification efficiency measurement of PCoV-Kit and LCoV-Kit. (XLSX)

S5 Table Raw data of clinical specimens. (XLSX)

S6 Table Statistics of 376 oropharyngeal swab specimens that tested positive with both LCoV-Kit and PCoV-Kit. (XLSX)

S7 Table Statistics of 64 oropharyngeal swab specimens with inconsistent test results. (XLSX)

S8 Table List of discrepancies in test results. (XLSX)

S1 Appendix (DOCX)
