# Supplementary material for: Evaluation of a novel lyophilized-pellet-based 2019-nCoV nucleic acid detection kit for the diagnosis of COVID-19
Source: PLoS One. 2023 Oct 25;18(10):e0292902. doi: 10.1371/journal.pone.0292902 (PMC10599558; doi:10.1371/journal.pone.0292902)
Supplement: S1 Appendix — (DOCX) [file pone.0292902.s009.docx]

# Evaluation of a novel lyophilized-pellet-based 2019-nCoV nucleic acid detection kit for the diagnosis of COVID-19

Yiyuan Xu^1^, Tian Xu^1^, Shaoting Chen^2^, Huakang Yao^3^, Yuxiang Chen^1^, Yanfen Zeng^4^, Falin Chen^2*^, Guanbin Zhang^1,5,6*^

^1^ Research and Development Department, Fujian CapitalBio Medical laboratory, Fuzhou, Fujian, China.

^2^ Clinical Laboratory, Fujian Provincial Hospital, Fuzhou, Fujian, China

^3^ Medical Department, Fujian Provincial Yongtai County Hospital, Fuzhou, Fujian, China

^4^ Fujian Provincial Center for Clinical Laboratory, Fujian Provincial Hospital, Fuzhou, Fujian, China

^5^ Research and Development Department, National Engineering Research Center for Beijing Biochip Technology, Beijing, China

^6^ Department of Laboratory Medicine, Fujian Medical University, Fuzhou, Fujian, China

^*^Corresponding authors

E-mail: [falinchen@126.com](mailto:falinchen@126.com) (FC), [gbzhang@capitalbio.com](mailto:gbzhang@capitalbio.com) (GZ)

**S1 Appendix**

**Specificity evaluation**

According to the instructions provided for LCoV-Kit and PCoV-Kit, it is explicitly mentioned that these kits do not exhibit any cross-reactivity with diverse respiratory viruses, bacteria, fungi, etc. In our data, these kits were validated to exhibit no cross-reactivity against specific respiratory pathogens, ensuring their specificity.

The respiratory viruses, including influenza A virus, influenza B virus, respiratory syncytial virus, human adenovirus, and human rhinovirus, were obtained from clinical oropharyngeal swabs. The respiratory bacteria, including *Acinetobacter baumannii*, *Mycoplasma pneumoniae*, *Escherichia coli*, *Stenotrophomonas maltophilia*, *Pseudomonas aeruginosa*, and *Haemophilus influenzae,* were obtained from sputum cultures. In addition, we continuously diluted the SARS-CoV-2 RNA reference material to a concentration below LOD (20 copies/mL) in order to assess the specificity of both LCoV-Kit and PCoV-Kit. The nucleic acid of respiratory viruses was extracted using nucleic acid (DNA/RNA) extraction or purification kit (Sansure Biotech, China, Cat. #SE40015), while the nucleic acid of respiratory bacteria was extracted using nucleic acid (DNA/RNA) extraction or purification kit (CapitalBio Genomics, China, Cat. # S10040). The extracted nucleic acids were amplified using LCoV-Kit and PCoV-Kit, respectively, following the protocols described in "Nucleic acid extraction and PCR amplification".

The results implied that LCoV-Kit and PCoV-Kit for SARS-CoV-2 had no cross-reactivity with other respiratory pathogens, while the primer/probe group exhibited no self-reactivity (S1 and S2 Figs).


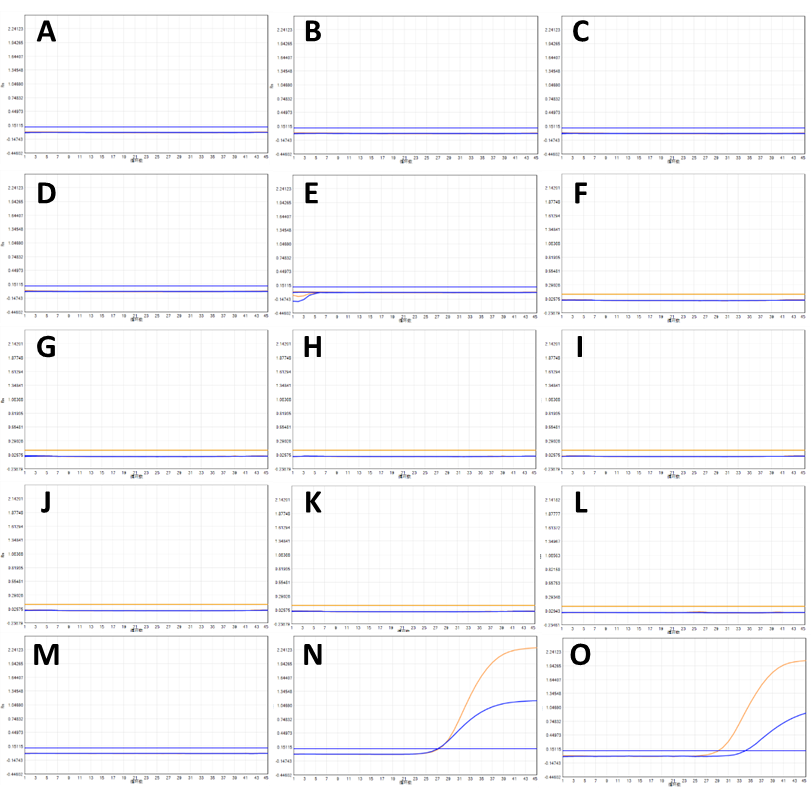


**S1 Fig. Amplification curves of LCoV-Kit.** (A) *Acinetobacter baumannii*; (B) *Escherichia coli*; (C) *Stenotrophomonas maltophilia*; (D) *Pseudomonas aeruginosa*; (E) *Haemophilus influenzae*; (F) respiratory syncytial virus; (G) *Mycoplasma pneumoniae*, (H) human adenovirus; (I) influenza A virus; (J) influenza B virus; (K) human rhinovirus; (L) SARS-CoV-2 RNA reference material diluted to 20 copies/mL; (M) negative control; (N) positive control; (O) borderline positive control.


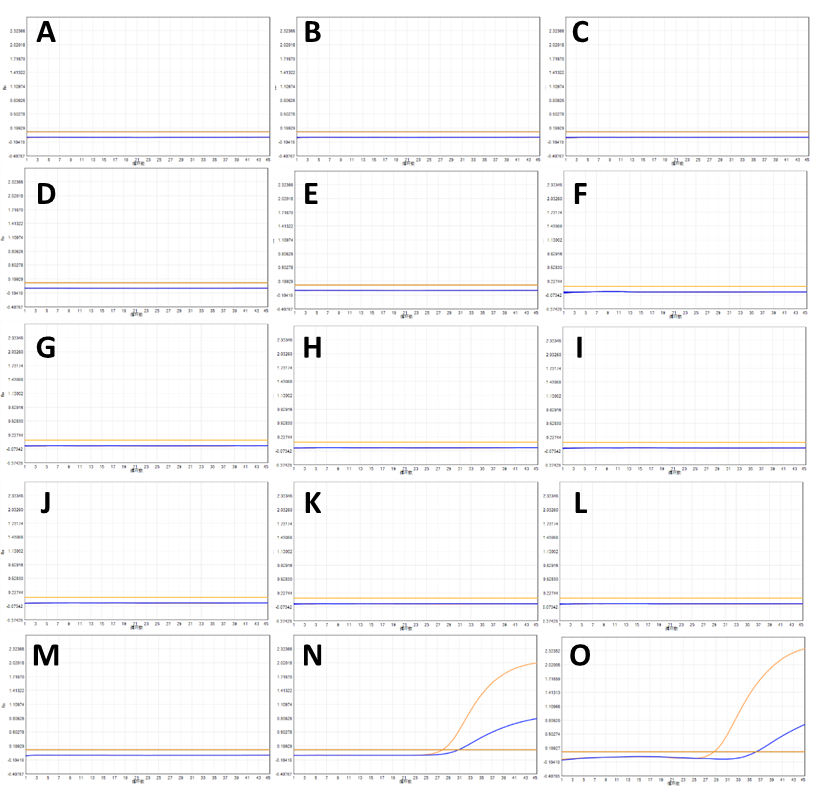


**S2 Fig. Amplification curves of PCoV-Kit.** (A) *Acinetobacter baumannii*; (B) *Escherichia coli*; (C) *Stenotrophomonas maltophilia*; (D) *Pseudomonas aeruginosa*; (E) *Haemophilus influenzae*; (F) respiratory syncytial virus; (G) *Mycoplasma pneumoniae*, (H) human adenovirus; (I) influenza A virus; (J) influenza B virus; (K) human rhinovirus; (L) SARS-CoV-2 RNA reference material diluted to 20 copies/mL; (M) negative control; (N) positive control; (O) borderline positive control.
